# Photovoltaic Performance Enhancement of Silicon Solar Cells Based on Combined Ratios of Three Species of Europium-Doped Phosphors

**DOI:** 10.3390/ma11050845

**Published:** 2018-05-18

**Authors:** Wen-Jeng Ho, Bang-Jin You, Jheng-Jie Liu, Wen-Bin Bai, Hong-Jhang Syu, Ching-Fuh Lin

**Affiliations:** 1 Department of Electro-Optical Engineering, National Taipei University of Technology, No. 1, Section 3, Zhongxial East Road, Taipei 10608, Taiwan; t105658002@ntut.edu.tw (B.-J.Y.); jjliu@mail.ntut.edu.tw (J.-J.L.); t105658023@ntut.edu.tw (W.-B.B.); 2 Graduate Institute of Photonics and Optoelectronics, National Taiwan University, No. 1, Section 4, Roosevelt Road, Taipei 10617, Taiwan; f98941054@ntu.edu.tw (H.-J.S.); lincf@ntu.edu.tw (C.-F.L.)

**Keywords:** Eu-doped phosphors, luminescent down-shifting (LDS), photovoltaic performance, silicon solar cells, spin-on film

## Abstract

This paper presents a scheme for the enhancement of silicon solar cells in terms of luminescent emission band and photovoltaic performance. The proposed devices are coated with an luminescent down-shifting (LDS) layer comprising three species of europium (Eu)-doped phosphors mixed within a silicate film (SiO_2_) using a spin-on film deposition. The three species of phosphor were mixed at ratios of 0.5:1:1.5, 1:1:1, or 1.5:1:0.5 in weight percentage (wt %). The total quantity of Eu-doped phosphors in the silicate solution was fixed at 3 wt %. The emission wavelengths of the Eu-doped phosphors were as follows: 518 nm (specie-A), 551 nm (specie-B), and 609 nm (specie-C). We examined the extended luminescent emission bands via photoluminescence measurements at room temperature. Closely matching the luminescent emission band to the high responsivity band of the silicon semiconductor resulted in good photovoltaic performance. Impressive improvements in efficiency were observed in all three samples: 0.5:1:1.5 (20.43%), 1:1:1 (19.67%), 1.5:1:0.5 (16.81%), compared to the control with a layer of pure SiO_2_ (13.80%).

## 1. Introduction

Solar energy is among the most promising forms of renewable energy [1,2]. Currently, wafer-based crystalline silicon solar cells are the mainstay of the photovoltaic industry, with a market share of approximately 85% of the production of photovoltaic devices worldwide [3,4]. The theoretical maximum conversion efficiency of crystalline silicon solar cells with band-gap energy of 1.1 V is just 31%, due to the effects of thermalisation [5], surface recombination [6,7], and spectral loss [8,9,10,11]. The conversion efficiency of crystalline silicon solar cells at ultraviolet-blue (UV–blue) wavelengths remains relatively low due to high surface recombination losses and low responsivity within the UV–blue wavelength band. Numerous methods have been devised to enhance conversion efficiency at short wavelengths, including the down-conversion (DC) or luminescent down-shifting (LDS) of the incident spectrum [12,13,14,15,16,17]. DC materials are able to convert high-energy incident photons into two or more photons of lower energy. The absorption of the resulting low-energy photons results in the generation of electron–hole pairs in the solar cell. This means that DC allows for the generation of more than one electron–hole pair from each high-energy photon. LDS has an effect similar to that of DC. However, only one photon is re-emitted from the luminescent converter. Europium-doped (Eu-doped) phosphors are ideally suited to LDS, due to their high quantum efficiency and large Stokes-shift [18,19]. In numerous studies, samples with single species of Eu-doped phosphors have been evaluated as an LDS material for photovoltaic devices [20,21,22,23,24]. However, few researchers have investigated combining multiple species of Eu-doped phosphors in various ratios [25,26].

In this study, we investigated silicon solar cells coated with an LDS layer comprising SiO_2_ with three species of Eu-doped phosphor in three weight percentages (wt %). We then examined the LDS effect and luminescent emission band via photoluminescence (PL) measurements at room temperature. We also used optical reflectance and external quantum efficiency to evaluate the effectiveness of LDS. Finally, we quantified the efficiency enhancement of cells with and without the coating of Eu-doped phosphors using photovoltaic current density-voltage (J–V) measurements.

## 2. Materials and Methods

### 2.1. Deposition and Characterization of LDS Layer Comprising Three Species of Eu-Doped Silicate Phosphor

Three species of Eu-doped silicate phosphor (InteMatix Company product, Fremont, CA, USA) are used for LDS spectral conversion. The fluorescence emission wavelengths of those Eu-doped phosphors were as follows: 518 nm (specie-A), 551 nm, (specie-B), and 609 nm (specie-C). The LDS layer was created by mixing Eu-doped phosphor powder in a silicate solution (Emulsitone Company product, Whippany, NJ, USA) and applying it via spin-on film deposition. Three LDS-layer samples were prepared with various combinations of the Eu-doped silicate phosphors, as follows: 1.5:1:0.5 (Sample-I), 1:1:1 (Sample-II), and 0.5:1:1.5 (Sample-III) in weight percentage (wt %). The total quantity of Eu-doped phosphor material mixed in the silicate solution was fixed at 3 wt % in all samples. The mixed solutions were spin-coated on clean silicon substrates at 3000 rpm for 60 s before being baked at 200 °C for 30 min under an air atmosphere. For comparison, we also fabricated samples with a SiO_2_ layer without Eu-doped phosphors using the same coating parameters. The surface morphology and chemical composition of the samples coated with Eu-doped phosphors LDS layer were examined using scanning electron microscopy and energy-dispersive X-ray spectroscopy (EDS; Hitachi S-4700, Hitachi High-Tech Fielding Corporation, Tokyo, Japan). The fluorescence emission band and LDS effects of the various samples (with single species or three species of Eu-doped phosphors) were examined via photoluminescence measurements (PL; Ramboss 500i Micro-PL Spectroscopy, DONGWOO Optron, Gyeonggi-Do, Korea) at room temperature using a solid-state UV laser with emission wavelength of 266 nm and emission power of 20 mW (MPL-F-266, Changchun New Industries Optoelectronics Technology Co., Ltd., Changchun, China) as a excitation source. The reflectance and light scattering effects of the silicate layer with and without Eu-doped phosphors particles was characterized using an UV–vis–NIR spectrophotometer (PerkinElmer LAMBDA 35, Waltham, MA, USA).

### 2.2. Fabrication and Characterization of Silicon Solar Cell Coated with LDS Layer Comprising Three Species of Eu-Doped Silicate Phosphor

**Figure 1:**
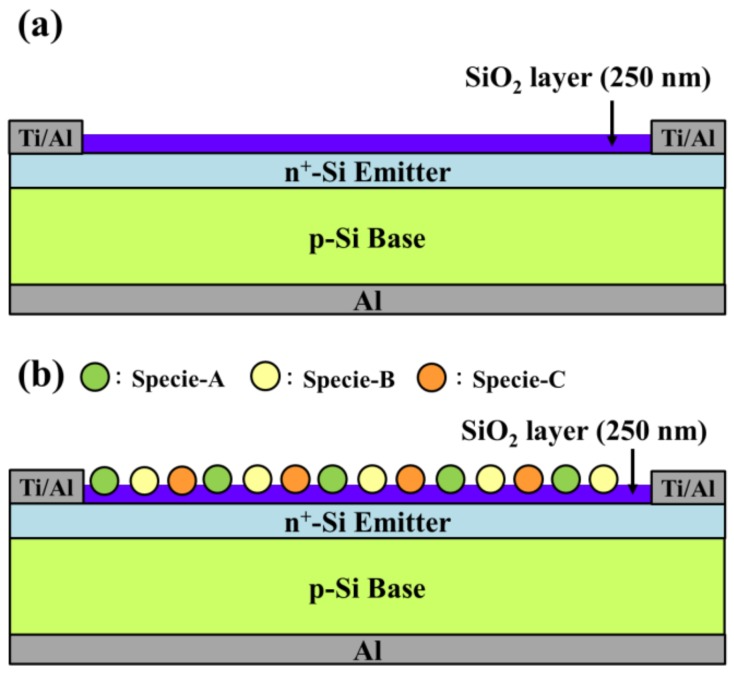
Schematic diagram showing silicon solar cell coated with (**a**) 250-nm-thick SiO_2_ layer; (**b**) luminescent down-shifting (LDS) layer (SiO_2_ layer comprising three species of Eu-doped silicate phosphors).

Figure 1 presents schematic diagrams of silicon solar cells coated with a SiO_2_ layer without Eu-doped phosphors and samples with an LDS layer of SiO_2_ with three species of Eu-doped silicate phosphor. A boron-doped crystalline silicon wafer with a thickness of 275 μm, (100) orientation, and resistivity of 10 Ω-cm was used as a starting material for the solar cell devices. After standard Radio Corporation of America (RCA) cleaning, an n^+^-Si emitter layer (300 nm-thick) was formed on the front-side of p-silicon via spin-on film processing using a liquid phosphorous source (Phosphorofilm, Emulsitone Co., Whippany, NJ, USA). The samples were then subjected to heat treatment in a rapid thermal annealing (RTA) chamber at 900 °C for 2 min under ambient N_2_. Following the formation of a diffuse n^+^-Si emitter layer, the phosphorous oxide remaining on the sample surface was etched using a buffered oxide etchant. Four-point probe resistivity and electrochemical capacitance-voltage profiling revealed that the n^+^-Si emitter layer had a sheet resistance of 50 Ω/sq and a peak phosphorus concentration of 1020 cm^−3^ at the surface. The samples were then divided into individual cells of 4 × 4 mm^2^ via isolation etching based on photolithography using a solution of HNO_3_:HF:H_2_O at a ratio of 1:1:2. Ohmic contact electrodes were produced by depositing an aluminum (Al) film to a thickness of 300 nm on the back side and a titanium (Ti)/Al film (20-nm-Ti/300-nm-Al) on the front side using e-beam evaporation. After metallization processing, the samples underwent thermal annealing in an RTA chamber under ambient N_2_ at 450 °C for 20 min to ensure good ohmic contact between the metallic electrodes and Si semiconductor. This completed fabrication of bare silicon solar cells (bare cells).

The external quantum efficiency (EQE; Enli Technology Co., Ltd., Kaohsiung City, Taiwan) response at wavelengths from 300 nm to 1100 nm was used to examine the LDS spectral conversion due to the multiple species of Eu-doped phosphor. Photovoltaic current density-voltage (J–V) measurements under one-sun air mass (AM) 1.5 G simulation (1000 mW/cm^2^ at 25 °C) were used to confirm the contribution of the LDS layer. The solar simulator (XES-151S, San-Ei Electric Co., Ltd., Osaka, Japan) was calibrated using a crystalline silicon reference cell (PVM-894, PV Measurements Inc., Boulder, CO, USA) certified by the National Renewable Energy Laboratory (NREL) prior to the measurement of devices.

## 3. Results and Discussion

**Figure 2:**
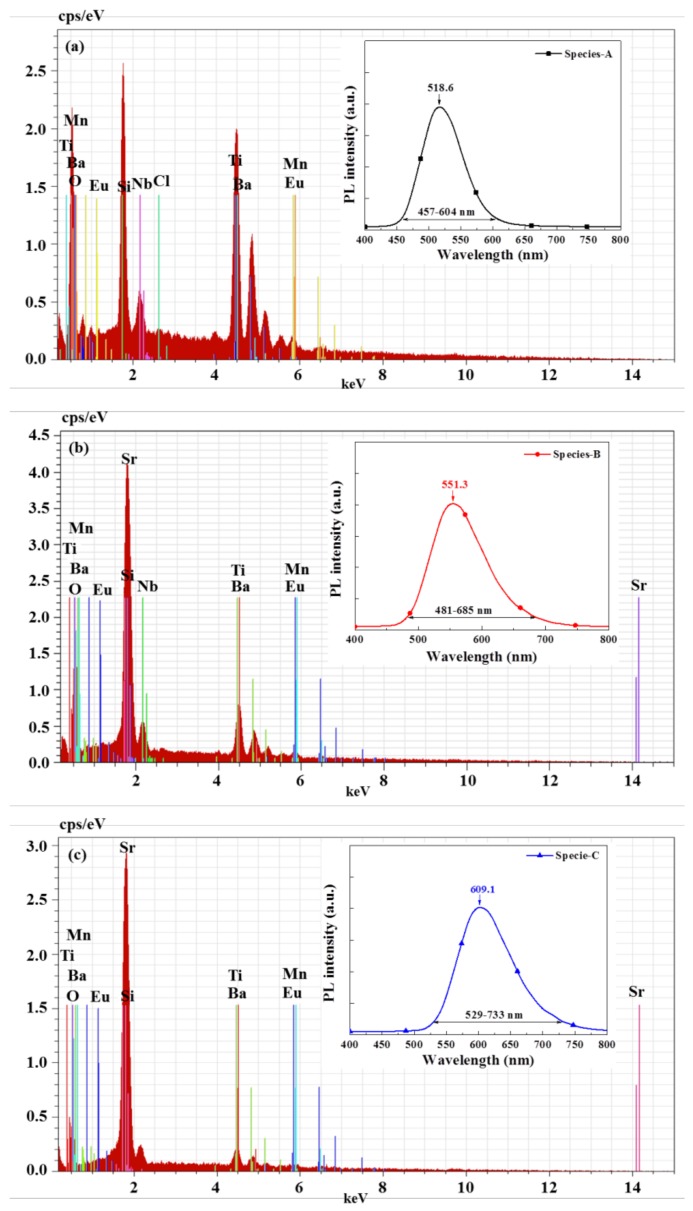
Energy-dispersive X-ray spectroscopy (EDS) spectra and fluorescence emission spectra of silicon samples coated with SiO_2_ layer comprising (**a**) specie-A; (**b**) specie-B; or (**c**) specie-C of Eu-doped silicate phosphors.

Figure 2 presents the EDS spectra and fluorescence emission spectra of silicon samples coated with a SiO_2_ layer comprising single (a) specie-A, (b) specie-B, and (c) specie-C, of Eu-doped silicate phosphors. EDS is used for element analysis and the chemical characterization of solid samples. Each element within the sample has a unique atomic structure, which produces a unique set of peaks in its electromagnetic emission spectrum. In this study, specie-A was primarily composed of O, Si, Ba, Ti, and Nb with small quantities of Eu, Mn, and Cl. Specie-B was primarily composed of O, Sr, Si, Ba, Ti, and Nb with small quantities of Mn and Eu. Specie-C was primarily composed of O, Sr, Si, Ba, and Ti with small quantities of Eu and Mn. PL measurements at room temperature revealed the following fluorescence emission peaks: specie-A (518.6 nm), specie-B (551.3 nm), and specie-C (609.1 nm). In the next section, the PL results of samples with a single species of Eu-doped silicate phosphor are compared to samples with a combination of species in various concentration ratios.

**Figure 3:**
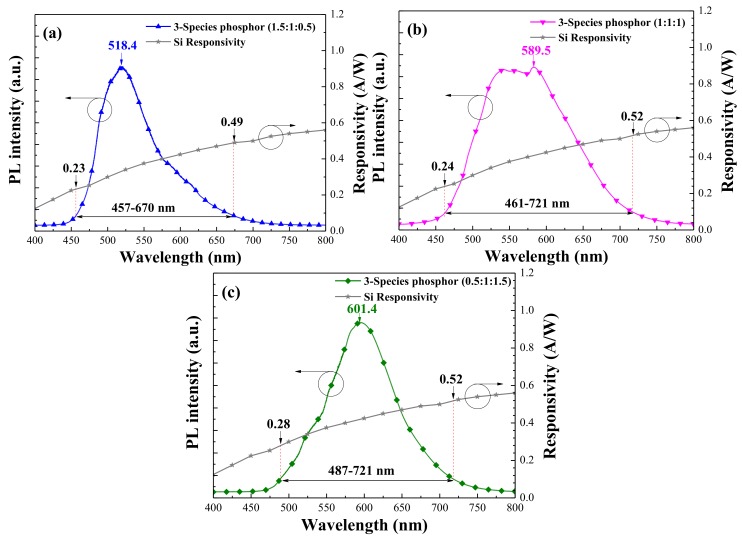
Photoluminescence (PL) emission spectra of LDS layer that included combinations of specie-A/specie-B/specie-C Eu-doped phosphors at (A:B:C) concentration ratios of (**a**) 1.5:1:0.5 (**b**) 1:1:1, and (**c**) 0.5:1:1.5 in wt %.

Figure 3 presents the PL emission spectra of the LDS layer containing a combination of specie-A/specie-B/specie-C Eu-doped phosphors at (A:B:C) concentration ratios of (a) 1.5:1:0.5, (b) 1:1:1, and (c) 0.5:1:1.5 in wt %. Figure 3 also plots the photo-responsivity of a bare silicon p-n junction device plotted for the sake of comparison. Table 1 lists the PL emission wavelength range, which is defined as the wavelength range at 10% of the maximum PL intensity corresponding to the responsivity values at these wavelength ranges. Figure 3a presents the band emission featuring a peak at 518.4 nm and a shoulder at approximately 580 nm in the sample with a phosphor combination of 1.5:1:0.5 in wt %. Figure 3b presents flat-band emission between 525 and 600 nm in the sample with a phosphor combination of 1:1:1. Figure 3c presents a similar band emission featuring a peak at 601.4 nm and a shoulder at approximately 540 nm in the sample with a phosphor combination of 0.5:1:1.5. The emission band of the LDS layer with a combination of three Eu-doped phosphor species was wider than that of the LDS layer with a single species. A wide emission band from the LDS layer with a combination of three Eu-doped phosphor species is highly beneficial to the re-emission of photons across a broad range of wavelengths for coupling with the active region of photovoltaic devices. The luminescent emission band of 1:1:1 and 0.5:1:1.5 samples extended into the high responsivity band of the silicon semiconductor. Thus, the wavelength of photons re-emitted from the 0.5:1:1.5 LDS layer approached a far higher responsivity region (0.28–0.52 A/W) and this responsivity region was higher than that of 0.24–0.25 A/W (1:1:1 LDS layer) and 0.23–0.49 A/W (1.5:1:0.5 LDS layer), thereby enhancing the conversion efficiency of the silicon solar cells. The broadband LDS performance of the cells was also confirmed using EQE and photovoltaic J–V measurements.

**Figure 4:**
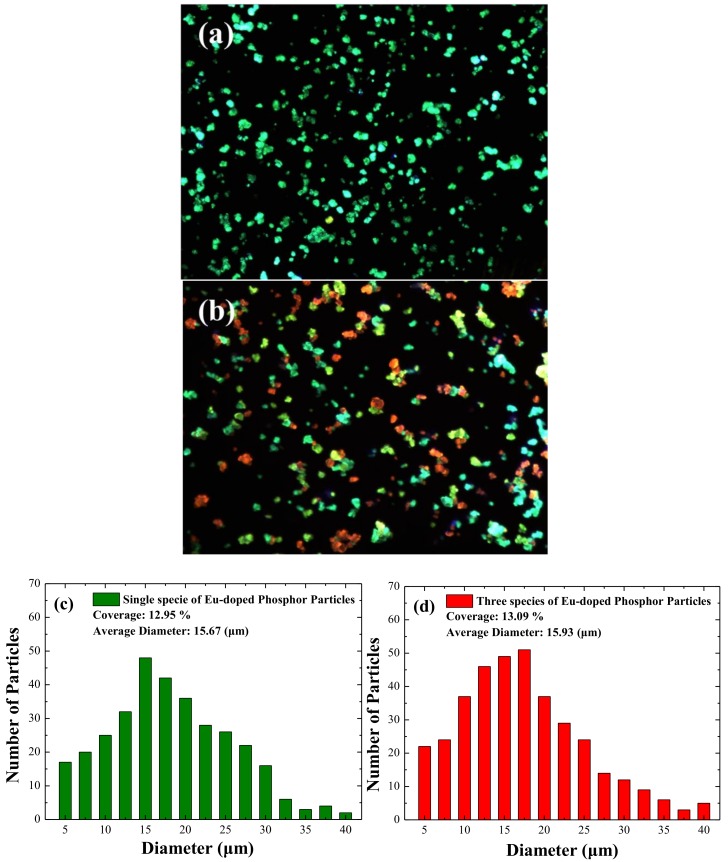
Optical microscope images (100×) of LDS layer with (**a**) single species (specie-A); (**b**) combination of specie-A/specie-B/specie-C; (**c**,**d**) size distribution and coverage of phosphor particles respectively calculated using Image-J software from images in Figure 4a,b.

Figure 4a,b present optical microscope images (100×) of LDS layers with (a) a single species (specie-A, in 3 wt %) and (b) a combination of specie-A/specie-B/specie-C at a concentration ratio of 1:1:1 in wt % under excitation by a 405-nm semiconductor laser. The intensity and color of photons re-emitted from the LDS layer can be clear observed. Figure 4c,d present the size distribution and coverage of the phosphor particles, which were, respectively, calculated using Image-J software from the images in Figure 4a,b. The phosphor particles had an average diameter of approximately 16 μm and a coverage of approximately 13%.

**Figure 5:**
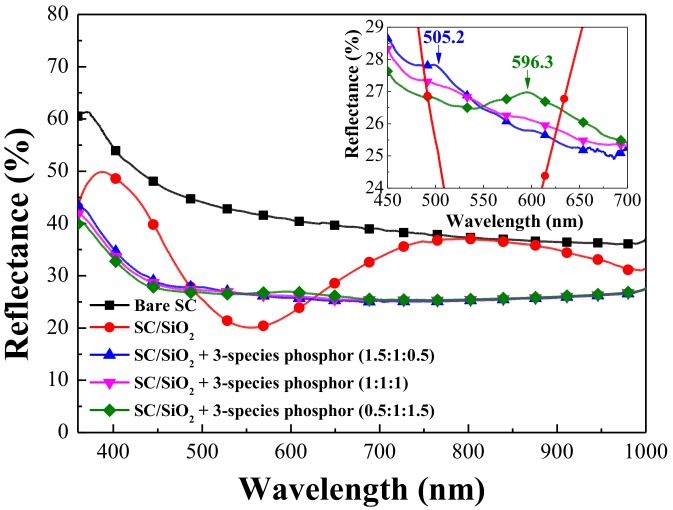
Reflectance spectra of bare silicon solar cell, cell coated with a 250-nm thick SiO_2_ layer, and cells coated with a SiO_2_ layer that included three species of Eu-doped phosphors in various weight ratios (0.5:1:1.5, or 1:1:1, or 1.5:1:0.5 in wt %).

Figure 5 presents the reflective spectra of the bare silicon solar cell, a cell coated with a 250-nm thick layer of SiO_2_, and cells coated with a SiO_2_ layer containing three species of Eu-doped phosphors at concentration ratios of 0.5:1:1.5, or 1:1:1, or 1.5:1:0.5 (wt %). The cell with a 250-nm thick SiO_2_ layer exhibited typical reflective characteristics, with lowest minimum reflectance of 19% at approximately 550 nm, due to destructive interference at the air/SiO_2_ and SiO_2_/Si interfaces. The reflectance of the cells coated with a SiO_2_ layer comprising Eu-doped phosphors was notably lower than that of the bare silicon solar cell at wavelengths from 350 to 1000 nm. The reflectance of the cells with Eu-doped phosphors was lower than that of cells with only a SiO_2_ layer (without Eu-doped phosphors) at a wavelength range of 350–500 nm and beyond 650 nm. The reduction in reflectance at 350–500 nm can be attributed to the absorption of incident photons by Eu-doped phosphors, whereas the effects beyond 650 nm can be attributed to the forward scattering of incident photons by phosphor particles on the surface. As shown in the inset in Figure 5, the reflectance between 500 and 625 nm was slightly higher due in part to the re-emission of photons from the Eu-doped phosphors and the less than ideal antireflection characteristics caused by non-uniformity in the thickness of the SiO_2_ layer because phosphor particles dispersed in the SiO_2_ layer. The inset in Figure 5 also reveals the increase in reflectance due to the re-emission of photons from the Eu-doped phosphors and the location of peak reflection values between wavelengths of 500 and 625 nm associated with the weight percentage of the phosphors. The LDS layers with wt % of 0.5:1:1.5 and 1.5:1:0.5 presented peak reflective wavelengths at 610 and 510 nm, which is in good agreement with the PL results. We also calculated the average weighted reflectance (*R_W_*) of all samples at wavelengths (*λ*) from 380 to 450 nm and from 380 to 1000 nm using Equation (1).

(1) $$R_{w}=\frac{\int_{\lambda_{\min}}^{\lambda_{\max}}R(\lambda )\cdot \varphi_{ph}(\lambda )d\lambda }{\int_{\lambda_{\min}}^{\lambda_{\max}}\varphi_{ph}(\lambda )d\lambda }$$

where *R*(*λ*) is reflectance as a function of wavelength and *φ_ph_*(*λ*) is the photon flux of AM 1.5 G solar energy spectrum as a function of wavelength. The resulting *R_W_* values are listed in Table 2. These results demonstrate that the average weighted reflectance of cells with Eu-doped phosphors was lower than that of cells with only a SiO_2_ layer (without Eu-doped phosphors) at a wavelength range of 380–450 nm and 380–1000 nm due to the effects of absorption and the forward scattering of incident photons by phosphor particles. The reduction of reflectance due to the SiO_2_ layer and the differently phosphor containing SiO_2_ layers would be correlated with the EQE increase.

**Figure 6:**
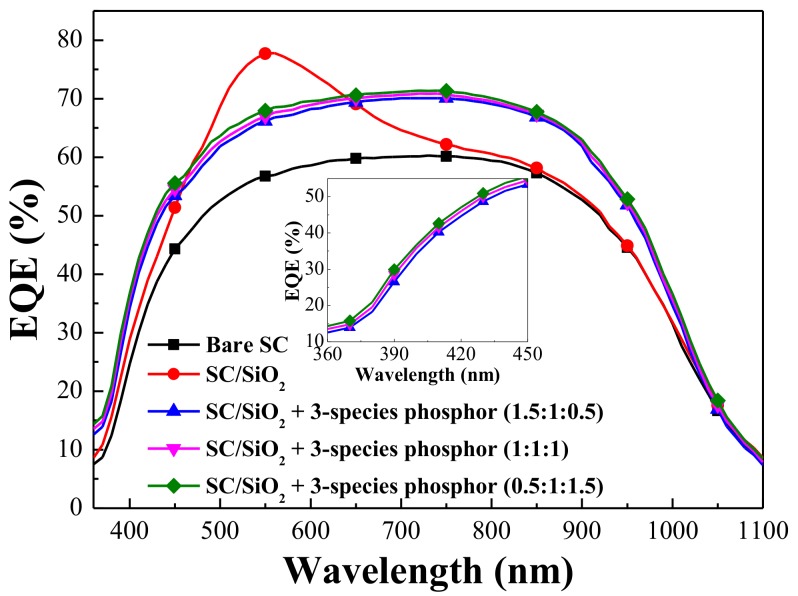
External quantum efficiency (EQE) response of bare silicon solar cell, cell coated with a 250-nm thick layer of SiO_2_, and cells coated with a SiO_2_ layer that included three species of Eu-doped phosphors at various weight ratios (0.5:1:1.5, or 1:1:1, or 1.5:1:0.5 in wt %).

Figure 6 presents the EQE response of the bare silicon solar cell, the cell coated with a 250-nm thick layer of SiO_2_, and cells coated with a SiO_2_ layer that included three species of Eu-doped phosphors in various weight ratios (0.5:1:1.5, or 1:1:1, or 1.5:1:0.5) (wt %). The EQE values of the cell with a 250-nm thick SiO_2_ layer were elevated at wavelengths of 350–900 nm due to the anti-reflective properties of the SiO_2_ layer. A peak EQE response of 78% was obtained at a wavelength of 550 nm, which is in good agreement with the optical reflectivity of the cell with only a SiO_2_ layer. The EQE response of cells with Eu-doped phosphors presented a broadband increase in EQE values from 350 to 1000 nm, compared to that of the bare silicon cell. Thus, the EQE response of the cells with Eu-doped phosphors is also in good agreement with the optical reflectance values. At wavelengths of 350–450 nm, the EQE values of the cells with Eu-doped phosphors were higher than that of the cell with only a SiO_2_ layer. This can be attributed to the LDS effects of the Eu-doped phosphor particles. Incident photons with wavelengths between 350 and 450 nm were absorbed by Eu-doped phosphor particles and re-emitted as photons at visible wavelengths (500–750 nm). This allowed the emitted photons to be absorbed near the depletion region of the p–n junction and also near the high responsivity band of the silicon solar cell. The resulting charge carriers provide higher photocurrent as well as higher collection efficiency. We also compared the average weighted EQE (*EQE_W_*) of cells from 380 to 450 nm and from 380 to 1000 nm. The *EQE_W_* results were calculated using Equation (2).

(2) $$EQE_{w}=\frac{\int_{\lambda_{\min}}^{\lambda_{\max}}EQE(\lambda )\cdot \varphi_{ph}(\lambda )d\lambda }{\int_{\lambda_{\min}}^{\lambda_{\max}}\varphi_{ph}(\lambda )d\lambda }$$

where *EQE*(*λ*) is the *EQE* value as a function of wavelength and *φ_ph_*(*λ*) is the photon flux of AM 1.5 G solar energy spectrum as a function of wavelength. The *EQE_W_* values are listed in Table 2. The *EQE* results demonstrate that the *EQE* of the 0.5:1:1.5 cell exceeded that of the 1:1:1 cell as well as that of the 1.5:1:0.5 cell. In addition, the *EQE* response of the cells with Eu-doped phosphors is in good agreement with the luminescence spectra of the respective phosphor mixtures.

**Figure 7:**
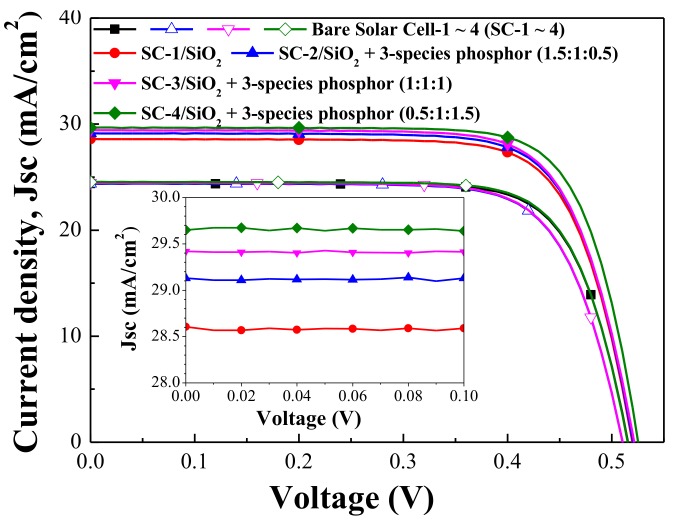
Photovoltaic J–V curves of bare silicon solar cell, cell coated with 250-nm thick layer of pure SiO_2_, and cells coated with SiO_2_ layer that includes three species of Eu-doped phosphor at various weight ratios (0.5:1:1.5, or 1:1:1, or 1.5:1:0.5 in wt %).

Figure 7 presents the photovoltaic J–V curves of the bare silicon solar cell, the cell coated with a 250-nm thick layer of SiO_2_, and cells coated with a SiO_2_ layer that included three species of Eu-doped phosphor at various weight ratios (0.5:1:1.5, or 1:1:1, or 1.5:1:0.5 in wt %). Table 3 details the photovoltaic performance of the cells. The short-circuit current density (J_sc_) and conversion efficiency (η) of the SiO_2_-coated cell and the cells with Eu-doped phosphors were higher than those of the bare solar cell. The J_SC_ of the cell with Eu-doped phosphors exceeded that of the cell with only SiO_2_, due to the additional LDS effects provided by forward-scattering by Eu-doped phosphor particles. The cell with Eu-doped phosphors (0.5:1:1.5 wt %) presented J_sc_ and η enhancements (∆J_sc_, ∆η) of 18.81% and 20.43% over the cell with a SiO_2_ layer. The cell with Eu-doped phosphors (1:1:1 wt %) presented J_sc_ and η enhancements of (∆J_sc_ = 17.70%, ∆η = 19.67%). The cell with Eu-doped phosphors (1.5:1:0.5) presented J_sc_ and η enhancements of (∆J_sc_ = 16.27%, ∆η = 16.81%). The improvement in photovoltaic performance in the 0.5:1:1.5 cells exceeded that of all other cells due to the wide-band luminescent emission and re-emission of incident photons into the high responsivity band of the silicon solar cell. In addition, the J_sc_ and η enhancements of the cells with Eu-doped phosphors are also in good agreement with the EQE response of the respective phosphor mixtures. We are currently engaged in optimizing the ratio of Eu-doped phosphors in order to maximize photovoltaic performance.

## 4. Conclusions

This paper describes efforts to enhance the photovoltaic performance of solar cells by coating them with a layer of SiO_2_ that includes three species of Eu-doped phosphor particles at various weight ratios. Compared to the cell coated with a layer of pure SiO_2_, we observed significant improvements in cell efficiency due to an increase in short-circuit current density and open-circuit voltage. This is an indication that the improvement in photovoltaic performance was due to wide-band luminescent emission and the forward scattering by the Eu-doped phosphor particles. It can also be attributed to the fact that the emission band of the LDS layer matched the high responsivity band of the silicon semiconductor. Compared to the bare solar cell, the efficiency enhancement of the cell with a species ratio of 0.5:1:1.5 wt % (20.43%) exceeded that of the cell with a species ratio of 1:1:1 wt % (19.67%) and the cell with a species ratio of 1.5:1:0.5 (16.81%).

## Figures and Tables

**Table 1:** Emission peaks, emission wavelength range at 10% of maximum PL intensity, and responsivity at the emission wavelength range of 10% of maximum PL intensity in all samples.

Combination of Eu-Doped Phosphors	PL Emission Peak (nm)	PL Emission Wavelength Range (nm) @ 10% of Max. Intensity	Responsivity (A/W) Range @ 10% of Max. Intensity
Specie-A	518.6	457–604	0.23–0.43
Specie-B	551.3	481–685	0.26–0.49
Specie-C	609.1	529–733	0.34–0.52
3-species phosphor (1.5:1:0.5)	518.4	457–670	0.23–0.49
3-species phosphor (1:1:1)	589.5	461–721	0.24–0.52
3-species phosphor (0.5:1:1.5)	601.4	487–721	0.28–0.52

**Table 2:** Average weighted reflectance (R_W_) and average weighted EQE (EQE_W_) calculated for wavelengths from 380 to 450 nm and from 380 to 1000 nm.

Silicon Solar Cell	R_W_ (%) @ 380–450 nm	R_W_ (%) @ 380–1000 nm	EQE_W_ (%) @ 380–450 nm	EQE_W_ (%) @ 380–1000 nm
Bare Solar Cell (SC)	51.69	41.23	32.77	50.51
SC/SiO_2_	45.03	31.69	37.58	58.09
SC/SiO_2_ + 3-species phosphor (1.5:1:0.5)	32.26	27.03	42.03	59.76
SC/SiO_2_ + 3-species phosphor (1:1:1)	31.52	26.79	43.36	60.31
SC/SiO_2_ + 3-species phosphor (0.5:1:1.5)	30.72	26.63	44.44	60.99

**Table 3:** Detailed photovoltaic performances of bare solar cell, cell with layer of pure SiO_2_, and cells with SiO_2_ layer that includes three species of Eu-doped phosphor at various weight ratios (0.5:1:1.5, or 1:1:1, or 1.5:1:0.5 in wt %).

Silicon Solar Cell	V_oc_ (mV)	J_sc_ (mA/cm^2^)	Fill Factor (%)	η (%)	ΔJ_sc_ (%)	Δη (%)
Bare Solar Cell-1 (SC-1)	518.4	24.56	73.45	9.35	---	---
SC-1/ SiO_2_	522.2	27.71	73.54	10.64	12.83	13.80
Bare Solar Cell-2 (SC-2)	518.1	24.39	73.47	9.28	---	---
SC-2/SiO_2_ + 3-species phosphor (1.5:1:0.5)	522.9	28.36	73.12	10.84	16.27	16.81
Bare Solar Cell-3 (SC-3)	517.8	24.47	73.04	9.25	---	---
SC-3/SiO_2_ + 3-species phosphor (1:1:1)	524.8	28.80	73.24	11.07	17.70	19.67
Bare Solar Cell-4 (SC-4)	517.7	24.34	73.04	9.20	---	---
SC-4/SiO_2_ + 3-species phosphor (0.5:1:1.5)	524.7	28.92	73.05	11.08	18.81	20.43
